# Dietary 5-Aminolevulinic Acid Alleviates Heat Stress-Induced Renal Injury in Laying Hens by Improving Mitochondrial Quality and Enhancing Antioxidant Activity

**DOI:** 10.3390/antiox14050556

**Published:** 2025-05-07

**Authors:** Fumika Nanto-Hara, Haruhiko Ohtsu

**Affiliations:** 1 Division of Meat Animal and Poultry Research, Institute of Livestock and Grassland Science, National Agriculture and Food Research Organization (NILGS), Tsukuba 305-0901, Japan; 2 Department of Research Promotion, Institute of Livestock and Grassland Science, National Agriculture and Food Research Organization (NILGS), Tsukuba 305-0901, Japan

**Keywords:** cGAS-STING, heat stress, heme oxygenase-1, laying hens, mitochondria, renal damage, 5-aminolevulinic acid

## Abstract

This study aimed to evaluate the effects of dietary 5-aminolevulinic acid (ALA) on laying hens to alleviate chronic heat stress-induced renal damage, resulting in improved egg productivity and eggshell quality. A total of 57 white-leghorn laying hens (46 weeks old) were randomly assigned to three groups and fed three experimental diets with different levels of ALA (0, 10, and 100 ppm) for 1 week. The birds in each group were then divided into two subgroups; one of the two subgroups was subjected to heat stress (33 °C for 3 weeks), whereas the other group was maintained at 24 °C. Heat exposure significantly decreased the laying rate and eggshell strength and caused renal damage, whereas ALA supplementation alleviated heat-induced poor productivity and renal damage. ALA increased the renal mitochondrial DNA copy number and downregulated the expression of the cGAS-STING pathway-related genes in the kidneys of heat-stressed hens. Furthermore, ALA upregulated the renal expression levels of *NRF2* and *HO-1*, whereas it downregulated those of *NF-κB* and tended to decrease the content of TBARS in the kidney (*p* = 0.07). Dietary ALA confers a renal protective effect by reducing heat-induced mitochondrial damage and enhancing antioxidant activity, which may contribute to improved productivity under chronic heat stress.

## 1. Introduction

The threat posed by global warming due to climate change is intensifying, with projections indicating an increase in extreme heat days and extended heat waves [1]. Excessive increases in environmental temperatures (heat stress) pose significant problems to the poultry industry, affecting production efficiency, animal health, and sustainability [2]. Despite the development of mitigation and adaptation strategies, their effectiveness is limited, and the development of effective strategies is crucial.

Specifically, heat stress negatively affects laying hens’ performance, reducing feed consumption, decreasing plasma calcium and phosphorus concentrations, and increasing panting, resulting in lower egg production and eggshell qualities [3,4]. The kidneys regulate calcium and phosphorus metabolism [5], which is important for eggshell formation. Previously, we showed that chronic heat stress causes severe kidney damage in laying hens, which may contribute to poor productivity [6,7]. Further, we showed that such renal damage may be related to mitochondrial dysfunction and inflammatory responses via cyclic GMP-AMP synthase stimulator of interferon genes (cGAS-STING) pathway activation in the kidney [6]. Therefore, feeding substances with renal protective properties to laying hens exposed to heat stress may improve productivity and eggshell quality.

5-Aminolevulinic acid (ALA) is a common precursor of all tetrapyrrole compounds, including chlorophyll, heme, and vitamin B12 [8]. As ALA is an endogenous amino acid that is nontoxic to humans and animals and easily degraded in the environment, it is garnering attention as a pharmaceutical and livestock feed additive [9]. Recently, extensive research has been conducted on the use of systems metabolic engineering strategies to construct industrially competitive ALA-producing microbial strains [10,11]. ALA is a mitochondrial activator synthesized from glycine and succinate by mitochondrial ALA synthase [12]. ALA is important in aerobic energy metabolism because it is a component of heme and cytochrome c, part of the mitochondrial electron transfer system [13]. Previous studies conducted in mammalian systems have shown that ALA administration to mice increases mitochondrial electron transfer chain activity, mtDNA copy number, and ATP levels [14,15]. Further, in both in vitro and in vivo studies in mammals, ALA treatment exerted a renoprotective effect by inhibiting nephrotoxicity and apoptosis by inducing heme oxygenase-1 (*HO-1*) expression [16,17]. HO-1 is an enzyme that regulates inflammatory responses and protects against oxidative stress in tissue injury [18].

Therefore, we hypothesized that dietary ALA supplementation in laying hens could alleviate heat stress-induced renal damage, resulting in improved egg productivity and eggshell quality. To verify these hypotheses and elucidate the underlying mechanism, we investigated the effects of dietary ALA supplementation on productivity and renal function in heat-stressed laying hens. In addition, we have examined the effects of ALA supplementation on renal mitochondrial damage and activation of the cGAS-STING pathway to determine the underlying mechanisms.

## 2. Materials and Methods

### 2.1. Ethics Statement

All procedures were approved by the Animal Care Committee of the Institute of Livestock and Grassland Science, National Agriculture and Food Research Organization, Japan (Approval number: 21C118ILGS), and in accordance with the ARRIVE guidelines. All experiments were performed in accordance with the relevant guidelines and regulations.

### 2.2. Animals, Diet, and Experimental Design

Animal experiments were performed as described in previous reports [6,7]. The experiment comprised a 4-week preliminary breeding period (adaptation) and a 4-week experimental period. Hens were raised individually in wire-floored cages (measuring 33 × 45 × 40 cm^3^ [width × height × depth]) with free access to feed and fresh water. During the preliminary breeding period, the egg production of 64 hens was recorded. Of those, 57 white-leghorn laying hens (46 weeks old, peak time of egg laying) weighing approximately 1590 ± 20.1 g, with the same feed consumption and egg production status (average laying rate: 95.1%), were used in this experiment. After the preliminary breeding period, birds were randomly divided into three groups with 19 hens each. The birds in each group were fed a corn–soybean meal-based diet (Crude protein: 15.5%; Metabolizable energy: 2.8 Mcal/kg; Calcium: 3.3%; Vitamin D: 500 IU/kg) supplemented with ALA (0, 10, or 100 ppm) designed to meet the Japanese feeding standard for poultry [19]. ALA was purchased from Biosynth Ltd. (Newbury, UK). After one week, the birds in each group were randomly divided into two subgroups (n = 10 or 9), one of which per main group (n = 9) was subjected to heat stress in a controlled environment chamber at 32 °C and 55% ± 5% relative humidity for 3 weeks, while the other was maintained at 24 °C and 55% ± 5% relative humidity (n = 10). The light regimen was 14 L:10 D, and the dark period went from 19:00 h to 05:00 h. The egg number and egg weight of each laying hen were recorded daily throughout the experiment, and the laying rate, average egg weight, and average daily egg production were calculated weekly. Once a week, eggs were collected to determine eggshell breaking strength and thickness and egg weight using a digital egg tester (Model DET6500, NABEL Co., Ltd., Kyoto, Japan). Body weight and feed intake were recorded once a week and on the final day of the experimental period. All birds were euthanized via rapid decapitation followed by exsanguination using appropriate equipment according to the procedures described in the *American Veterinary Medical Association Guidelines for the Euthanasia of Animals: 2020 Edition* [20]. Renal tissue samples from all birds were immediately stored at −80 °C or in RNAlater (Thermo Fisher Scientific, Waltham, MA, USA) at −20 °C until further use.

### 2.3. Blood Collection and Analysis

On the final day of the experimental period, blood samples were collected from all birds by venipuncture from the branchial vein. Blood samples were centrifuged at 3000× *g* for 15 min at 10 °C to separate the plasma from the whole blood. Plasma was then transferred into 1.5 mL labeled vials and stored at −20 °C until further use. Plasma concentrations of calcium, phosphate, blood urea nitrogen, creatinine, and albumin were analyzed by Kotobiken Medical Laboratories (Ibaraki, Japan).

### 2.4. Histological Analysis of the Fibrotic Area

Renal tissues were fixed in 4% paraformaldehyde, dehydrated in a graded series of 70%, 80%, 90%, 95%, and 100% ethanol, and embedded in paraffin. Then, paraffin-embedded tissues were cut into 4 μm sections, deparaffinized in xylene and hydrated in a graded series of ethanol–water mixture, and stained with Masson’s trichrome. Light microscopy (Leica, Nusslock, Germany) was used to observe histopathological changes in the kidney. Eight fields per sample were randomly selected for fibrotic area quantification using ImageJ software version 6.0 (Media Cybernetics, Inc., Rockville, MD, USA). Images were acquired at the same magnification under identical conditions, and the fibrotic area was expressed as a percentage of the captured image area.

### 2.5. Total RNA Isolation, cDNA Synthesis, and Real-Time PCR

Total RNA from kidney samples was extracted using the RNeasy Mini Kit (Qiagen, Venlo, The Netherlands) according to the manufacturer’s instructions. Complementary DNA (cDNA) was synthesized from 1 μg of total RNA using random primers (TOYOBO, Tokyo, Japan) and ReverTra Ace (TOYOBO). Real-time PCR was performed in duplicate to measure mRNA expression using a QuantStudio 5 Real-time PCR system (Applied Biosystems, Foster City, CA, USA) and THUNDERBIRD SYBR qPCR Master Mix (TOYOBO, Tokyo, Japan). Table 1 lists the primer sequences for the target and reference genes. PCR primers for chicken nuclear factor (NF)-κB were purchased from Qiagen (Venlo, The Netherlands).

### 2.6. Determination of 2-Thiobarbituric Acid Reactive Substances (TBARS) Content

Lipid peroxidation in renal tissue was measured using the TBARS assay [25]. Frozen kidney tissues (100 mg) were homogenized in 0.9 mL of ice-cold potassium chloride buffer (1.15%, *w/v*) by bead beating at 2000× *g* for 30 s using a Cell Destroyer (Pro Sense Inc., Tokyo, Japan). In brief, the reaction mixture contained 1.5 mL acetic acid (20%, *w/v*, pH 3.5), 1.5 mL 2-thiobarbituric acid (0.8% *w/v*), 0.2 mL sodium dodecyl sulfate (8.1%, *w/v*), 0.3 mL distilled water, and 0.2 mL tissue homogenate. The mixture was incubated at 95 °C for 60 min. Samples were then cooled and extracted into a 2.5 mL mixture of n-butanol and pyridine (15:1, *v/v*) and 0.5 mL distilled water. After centrifugation, the organic layer was collected, and the fluorescence was measured at 532 nm (Shimadzu UV-1800; Shimadzu, Kyoto, Japan) and quantified as the malondialdehyde equivalent. Triglyceride (TG) concentrations of homogenate samples were determined using the WAKO LabAssay TG Kit (Wako Pure Chemical, Osaka, Japan) according to the manufacturer’s instructions. Each assay was performed in duplicate.

### 2.7. Tissue ATP Content

Cellular ATP in the kidney tissue was measured using the ATP assay kit (TA100, Toyo B Net). Briefly, small pieces of tissue (approximately 0.1 g) were washed once with PBS, resuspended in ATP extraction reagent, and centrifuged at 1000× *g* for 10 min. Then, the supernatant was used for the ATP assay. Next, the ATP levels were quantified using an ATP assay kit with luciferin and luciferase according to the manufacturer’s (Toyo B Net, Tokyo, Japan) instructions. The protein concentrations of homogenate samples were determined via the BCA assay using the TaKaRa BCA protein assay kit (Takara Bio Inc., Shiga, Japan). Each assay was performed in duplicate.

### 2.8. Determination of mtDNA Relative Expression and Copy Number

mtDNA was extracted from kidney samples using the DNeasy blood and tissue kit (Qiagen, Venlo, The Netherlands) per the manufacturer’s instructions. All steps were completed at room temperature (22–24 °C), and each sample was processed individually. The relative mtDNA expression was measured using THUNDERBIRD SYBR qPCR Master Mix (TOYOBO, Tokyo, Japan). The primer sequences for the target and reference genes are shown in Table 2. The mtDNA copy number was determined using the following equation:

copies=2^ (−Ct mt)/(−Ct reference)

### 2.9. Statistical Analysis

Statistical analysis was performed using R software (ver. 4.4.2). Data were analyzed using a two-way, and the means were compared using Tukey’s multiple comparison post hoc test. Differences were considered significant at *p* < 0.05 and to indicate a trend at 0.05 ≤ *p* < 0.1.

## 3. Results

### 3.1. Laying Performance and Eggshell Quality

As shown in Table 3, chronic heat exposure significantly decreased body weight, feed intake, laying rate, egg weight, and daily egg production, as well as eggshell strength, weight, and thickness (*p* < 0.05). The laying rate was significantly affected by ALA supplementation, and there was an interaction of temperature and ALA on the laying rate (*p* < 0.05). Under chronic heat stress, the highest laying rate and daily egg production were observed when hens were supplemented with 100 ppm ALA. Exposure of control diet-fed hens to heat stress resulted in a significantly decreased eggshell strength compared with thermoneutral controls. Under chronic heat stress, the decrease in eggshell strength significantly improved with the addition of 10 ppm ALA to the diet.

### 3.2. Biochemical Plasmatic Parameters

As shown in Table 4, heat exposure significantly affected creatinine, albumin, calcium, and phosphate concentrations in plasma. ALA supplementation tended to reduce the plasma concentrations of creatinine (*p* = 0.06). No temperature × diet interaction was observed for plasma biochemical parameters.

### 3.3. Histological Analysis of the Fibrotic Area and Profibrotic mRNA Expression in Renal Tissue

**Figure 1:**
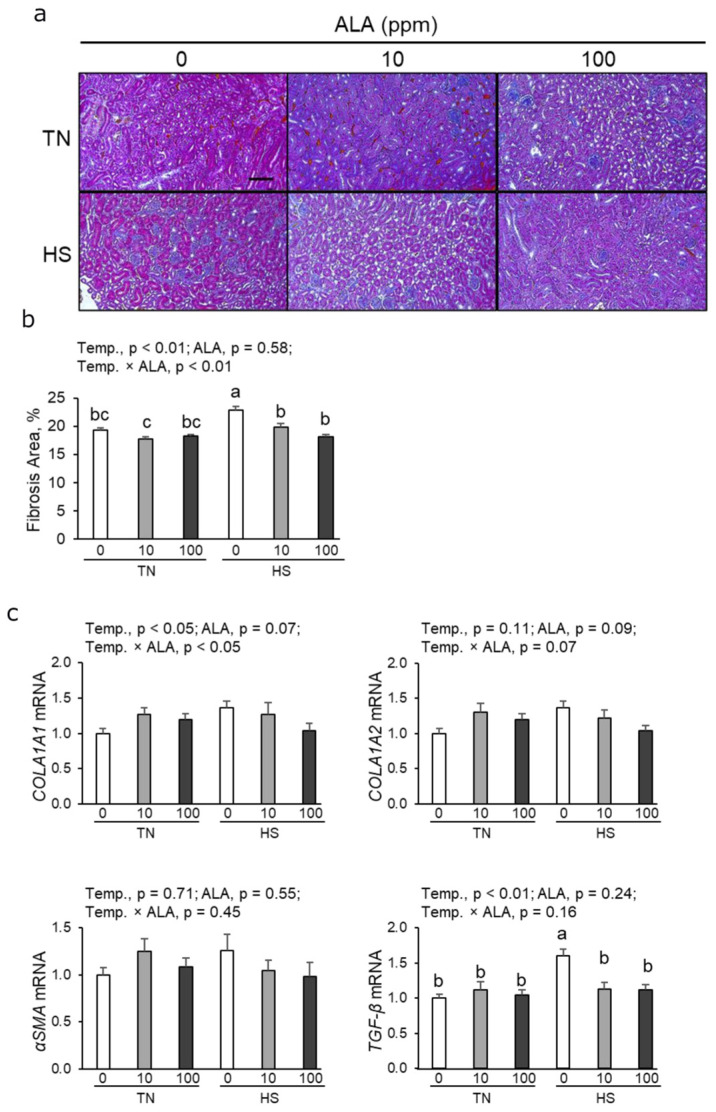
Effect of dietary ALA supplementation on renal fibrosis area and profibrotic mRNA expression in laying hens under chronic heat stress conditions. (**a**) Representative Masson’s trichrome-stained sections of hen kidneys. Bars = 100 µm. (**b**) Morphometric analysis of the fractional cortical tubular area of Masson’s trichrome-stained kidney images. n = 9–10 for each group. Data are presented as the percentage of fibrotic/nonfibrotic area of the renal cortex and mean ± SEM. Differences between groups were indicated by different lowercase letters (*p* < 0.05). (**c**) mRNA expression levels of profibrotic genes (*COLA1A1*, *COLA1A2*, *αSMA*, and *TGF-β*). Gene expression levels were quantified by real-time quantitative PCR and normalized to 18srRNA. n = 9–10 in each group. Data were presented as means with SEM. Abbreviations: TN, thermoneutral; HS, heat stress; ALA, 5-aminolevulinic acid; Temp, temperature; COL1A1, collagen Type I Alpha 1; COL1A2, collagen Type I Alpha 2; αSMA, α-smooth muscle actin; TGF-β, transforming growth factor-β.

To observe the effects of ALA on chronic heat stress in the kidney, we acquired histological images of the renal cortex. As shown in Figure 1a,b, the fibrotic area in the renal cortex significantly increased in heat-stressed hens. Both 10 and 100 ppm ALA supplementation significantly decreased the renal fibrotic area under heat stress (Figure 1b). Heat exposure increased the expression levels of collagen Type I Alpha 1 (*COLA1A1*) and transforming growth factor-β (*TGF-β*); however, it did not significantly affect the levels of collagen Type I Alpha 2 (*COLA1A2*) and α-smooth muscle actin (*αSMA*) (Figure 1c). Under heat stress, dietary supplementation with both 10 ppm and 100 ppm ALA significantly decreased TGF-β expression (Figure 1c).

### 3.4. Oxidative Damage, ATP Content, and mtDNA Copy Number in Renal Tissue

**Figure 2:**
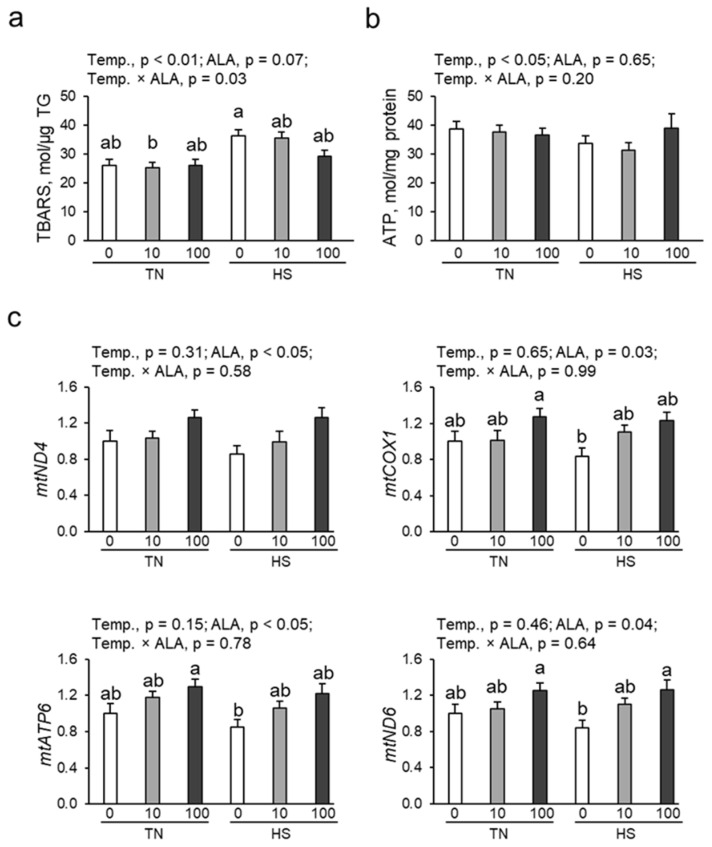
Effect of dietary ALA supplementation on oxidative damage, ATP contents, and mtDNA copy number in the renal tissue of laying hens under chronic heat stress. (**a**) TBARS content, (**b**) ATP content, and (**c**) mtDNA copy number in renal tissues of laying hens. n = 9–10 in each group. Data were presented as means with SEM. Differences between groups were indicated by different lowercase letters (*p* < 0.05). mt/nucDNA = mtDNA relative to nuclear DNA (β-actin) copy number. Abbreviations: TN, thermoneutral; HS, heat stress; ALA, 5-aminolevulinic acid; Temp, temperature; TBARS, 2-thiobarbituric acid reactive substances; TG, triglyceride; mtDNA, mitochondrial DNA; ND4, NADH dehydrogenase subunit 4; COX1, mitochondrial cytochrome c oxidase 1; ATP6, ATP synthase F0 subunit 6; ND6, NADH dehydrogenase subunit 6.

The renal contents of TBARS (a marker of oxidative damage) and ATP were determined. As shown in Figure 2a, heat exposure significantly increased the renal TBARS content, whereas ALA supplementation tended to decrease the TBARS content in the kidney (*p* = 0.07). Heat exposure significantly decreased ATP contents in renal tissue, which ALA supplementation could not improve (Figure 2b). Quantitative PCR analysis was used to analyze the mtDNA copy number in the renal tissue of laying hens based on mitochondrial *ND4*, *COX1*, *ATP6*, and *ND6* (Figure 2c). ALA supplementation significantly increased mtDNA copy numbers (*ND4*, *COX1*, *ATP6*, and *ND6*). No temperature × ALA interaction was observed for mitochondrial parameters.

### 3.5. Expression of Genes Related to the cGAS-STING Pathway in Renal Tissue

**Figure 3:**
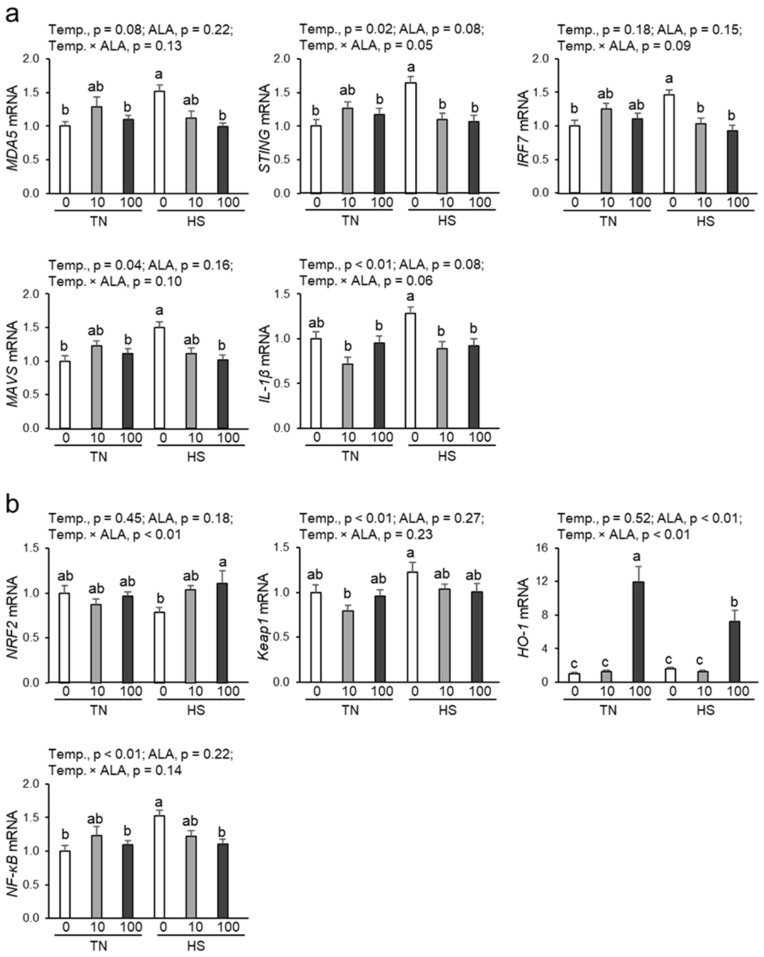
Effect of dietary ALA supplementation on the expression of genes related to the cGAS-STING and NRF2/HO-1 pathways in the renal tissue of laying hens under chronic heat stress. (**a**) mRNA levels of cGAS-STING pathway-related genes (*MDA5*, *STING*, *IRF7*, *MAVS*, and *IL-1β*), and (**b**) mRNA levels of Nrf2/HO-1 pathway-related genes (*NRF2*, *Keap1*, *HO-1*, and *NF-kB*). mRNA expression was quantified by real-time quantitative PCR and normalized to 18srRNA. n = 9–10 in each group. Data were presented as means with SEM. Differences between groups were indicated by different lowercase letters (*p* < 0.05). Abbreviations: TN, thermoneutral; HS, heat stress; ALA, 5-aminolevulinic acid; Temp, temperature; MDA5, melanoma differentiation-associated gene 5; STING, stimulator of interferon genes; IRF7, type I interferon regulatory factor 7; MAVS, mitochondrial antiviral signaling; IL-, interleukin-; NRF2, nuclear factor erythroid 2-related 2; Keap1, Kelch-like ECH-associated protein 1; HO-1, heme oxygenase-1; NF-κB, nuclear factor-κB.

As shown in Figure 3a, the expression of *STING* and *MAVS*, genes related to the cGAS-STING pathway, significantly increased under heat stress. Similarly, the expression of the cGAS-STING pathway-related gene *MDA5* tended to increase with heat exposure (*p* = 0.08). Under heat stress, dietary ALA supplementation with both 10 and 100 ppm significantly suppressed *STING* and *IRF7* expression, while only 100 ppm ALA could suppress the upregulated *MDA5* and *MAVS* expression. Furthermore, expression of *IL-1β*, a proinflammatory cytokine, was significantly upregulated by heat stress, an effect significantly suppressed by dietary ALA supplementation at both 10 and 100 ppm.

### 3.6. Expression of Genes Related to the NRF2/HO-1 Pathway and NF-kB in Renal Tissue

As shown in Figure 3b, heat exposure significantly affected expression of Kelch-like ECH-associated protein 1 (*Keap1*) and *NF-kB*, but not that of nuclear factor E2-related factor 2 (*NRF2*) and *HO-1*. Dietary ALA supplementation dramatically increased *HO-1* mRNA expression but not that of *NRF2*, *Keap1*, and *NF-kB*. Under heat stress, the expression of *NRF2* and *HO-1* was significantly increased by 100 ppm ALA supplementation; however, it did not increase upon 10 ppm ALA supplementation. Moreover, under heat stress, *NF-kB* expression was significantly suppressed by 100 ppm ALA supplementation. No significant interaction between heat exposure and dietary ALA supplementation was observed for *Keap1*, *NRF2*, and *NF-kB* expression.

## 4. Discussion

ALA has been suggested as a novel feed additive for farm animals to enhance animal health and productivity due to its abundant nutrients and biological functions [28]. Dietary ALA supplementation has been reported to have a significant beneficial effect on iron status and immune response; however, little effect on growth performance in dairy cows, pigs, broiler chickens, and laying hens [29,30,31,32]. Thus far, little information is available on the effects of ALA on laying performance under heat stress. In the present study, we found that chronic heat exposure caused renal damage and decreased egg productivity and eggshell quality, which is consistent with previous reports by Nanto-Hara et al. [6,7]. Importantly, under heat stress, dietary ALA supplementation tended to attenuate renal injury.

The kidney is a mitochondria-rich organ that requires large amounts of energy to maintain homeostasis by ultrafiltrate reabsorption at the glomerulus [33,34]. Recent studies indicated a role of mitochondrial dysfunction in the development and progression of renal disease [35]. Mitochondrial dysfunction leads to increased oxidative stress, decreased ATP contents, and dysregulated mtDNA quality, resulting in renal dysfunction [36,37]. Accordingly, our previous study demonstrated that chronic heat exposure increased TBARS contents and decreased ATP contents and mtDNA copy number in the renal tissue of laying hens [6,7]. Similarly, in the present study, we found that chronic heat exposure induced oxidative stress and mitochondrial dysfunction in laying hen kidneys. Notably, dietary ALA supplementation tended to alleviate the oxidative damage and significantly improved the quality of mtDNA, but not ATP contents, in the kidneys of laying hens. Our findings are consistent with recent reports indicating that ALA treatment increased the relative mtDNA copy number by enhancing the mitochondrial DNA maintenance pathway and reducing mitochondrial oxidative damage by reactive oxygen species (ROS) removal and increased activity of antioxidant enzymes in a mouse model [15,38]. Another recent study has shown that ALA treatment effectively enhances the oxygen consumption rate and ATP contents in human fibroblasts [39]. However, in this study, ALA supplementation did not significantly improve the heat-induced ATP depletion in renal tissue caused by heat stress. In pathological renal failure, the decreased mtDNA quality may contribute to the activation of the cGAS-STING pathway and its downstream inflammatory response [40,41]. Therefore, the maintenance of mtDNA quality by ALA supplementation may suppress cGAS-STING pathway activation. Indeed, dietary ALA supplementation downregulated the expression of the cGAS-STING pathway-related genes (*MDA5*, *STING*, *IRF7*, *MAVS*, and *IL-1B*) in the kidneys of heat-stressed hens. Thus, these results indicate that dietary ALA supplementation has a renoprotective effect by alleviating heat-induced mitochondrial damage and inhibiting the activation of the cGAS-STING pathway.

To provide evidence of a possible mechanism underlying the renoprotective effects associated with ALA supplementation, in this study, we examined the expression of *HO-1*, a well-known cytoprotective factor against oxidative stress and inflammation. ALA upregulates *HO-1* expression in renal cells, and that increased *HO-1* expression protects against kidney injury through antioxidant properties and by suppressing proinflammatory cytokines [42,43,44]. Similarly, HO-1 attenuates interstitial fibrosis and apoptosis in cyclosporine nephropathy [17]. Our results showed that dietary ALA supplementation upregulated HO-1 expression, particularly at 100 ppm. HO-1 is regulated through NRF2 and Keap1 [45]. Under basal conditions, NRF2 is bound to Keap1, and upon stimulation by ROS or other factors, it translocates to the nucleus, activating antioxidant-responsive genes such as *HO-1* [46]. In the present study, while no significant changes were observed in Keap1 expression, 100 ppm ALA supplementation upregulated *NRF2* expression under heat stress but not under thermoneutral conditions. Furthermore, the addition of 100 ppm ALA significantly reduced *NF-κB* expression only under heat stress. Heat exposure elevates ROS production and induces the upregulation of *NRF2* and *HO-1* expression [21,47], which is increased by ALA supplementation [48]. It has been reported that the antioxidant effect of ALA induces mild oxidative stress and preconditions cells against ROS [42,49]. Specifically, ROS induction supports the generation of antioxidants by activating both *NRF2* and *HO-1* and inhibits oxidative stress by suppressing *NF-κB* [42,49]. This antioxidant effect of ALA contributes to reduced TBARS content in the kidneys of heat-stressed hens supplemented with ALA. Thus, ALA supplementation, especially at 100 ppm, enhances *HO-1* expression via *NRF2* activation and suppresses *NF-κB* expression; its antioxidative properties would then inhibit heat-induced oxidative damage and exert renoprotective effects in heat-stressed laying hens.

There are several limitations of this study. First, although ALA affects mitochondria in the kidney and organs throughout the body, the effects on other organs such as the liver and digestive tract were not examined. To clarify the effects of ALA supplementation on laying hens under heat stress conditions, investigating the interrelationships between the kidney and other organs is necessary. Second, the heat stress model used herein does not completely reflect the diurnal pattern in summer, and the experimental period is short, and the experimental scale is small. Thus, the beneficial effect of dietary ALA supplementation on improving the renal injury and productivity of laying hens should be further verified at the poultry farm scale during the high-temperature summer season.

## 5. Conclusions

Dietary ALA supplementation protects against heat stress-induced renal injury in laying hens, which may contribute to the improvement in the low productivity observed under chronic heat stress conditions. This beneficial effect of ALA is dependent on suppressing the cGAS-STING pathway through the maintenance of mitochondrial quality and antioxidant effects through the enhancement of the NRF2/HO-1 pathway, both playing an important role in suppressing fibrosis and maintaining renal function. These findings emphasize the importance of mitochondrial quality control and reduction in oxidative damage as a crucial mechanism for the therapeutic effect of ALA in alleviating heat stress-induced renal injury.

## Figures and Tables

**Table 1:** Sequences of primers used for quantitative real-time PCR.

Gene	Primer Sequences (5′–3′)	Accession No.	Source or Reference of the Primer Sequences
*COL1A1*	F: ACCTCAGCAAGAACCCCAAG	XM_025144131.2	[21]
	R: CTCACCGCCGTACTCAAACT		
*COL1A2*	F: GCGGTTTCTACTGGATTGA	NM_001079714.2	[21]
	R: AGCGAGACGGCTTATTTG		
*αSMA*	F: AAGCACCACTGAATCCCAAAG	NM_001031229.1	[21]
	R: CCAGAGTCAAGCACAATCCCT		
*TGF-β*	F: GCAAACTGCGTCTGACCG	NM_001318456.1	[21]
	R: ACGAAGAAGATGCTGTGGC		
*MDA5*	F: CGAATGAAAACCTGGGACAG	AB371640	[22]
	R: TGGTTTTGCCACTGCCTGTA		
*STING*	F: CGGCTGTGACATCTGGGAT	KP893157	[22]
	R: CCCGAGTCAGGATGGTCTC		
*IRF7*	F: ACAACGCCAGGAAGGATGTC	NM_205372	[22]
	R: CCAGCAGCATGAACATGTGA		
*MAVS*	F: GAACGCAAACCACCTTCAAC	NM_001012893	[22]
	R: CCAGGAGCAGCACTCAAATC		
*IL-1β*	F: GGCCTGAGTCATGCATCGTT	NM_204524.1	[22]
	R: ATAAATACCTCCACCCCGACAA		
*NRF2*	F: GGACGGTGACACAGGAACAAC	NM_205117.1	[23]
	R: CTCCACAGCGGGAAATCAGAAAG		
*Keap1*	F: ACTTCGCTGAGGTCTCCAAG	NM_012289.4	[23]
	R: CAGTCGTACTGCACCCAGTT		
*HO-1*	F: AGCTTCGCACAAGGAGTGTT	NM_205344.2	[23]
	R: CTCCGAGTTCTCCCCGAAAG		
*18srRNA*	F: TCAGATACCGTCGTAGTTCC	HQ873432.1	[24]
	R: TTCCGTCAATTCCTTTAAGTT		

Abbreviations: COL1A1, collagen Type I Alpha 1; COL1A2, collagen Type I Alpha 2; αSMA, α-smooth muscle actin; TGF-β, transforming growth factor-β; MDA5, melanoma differentiation-associated gene 5; STING, stimulator of interferon genes; IRF7, type I interferon regulatory factor 7; MAVS, mitochondrial antiviral signaling; IL-, interleukin-; NRF2, nuclear factor E2-related factor 2; Keap1, Kelch-like ECH-associated protein 1; HO-1, heme oxygenase-1; 18srRNA, 18S ribosomal RNA; F, forward; R, reverse.

**Table 2:** Primer sequences used for mtDNA analysis.

Gene	Primer Sequences (5′-3′)	Accession No.	Source or Reference of the Primer Sequences
^1^ *ND4*	F: CGCAGGCTCCATACTACTCG	NC_040970.1	[26]
	R: TTAGGGCACCTCATAGGGCT		
^1^ *COX1*	F: CCATACTACTTACCGACCGCAACC	NC_040970.1	[27]
	R: GTGTCTACGTCCATTCCGACTGTG		
^1^ *ATP6*	F: ATTCTCAAGCCCCTGCCTAC	NC_053523.1	[27]
	R: TCAGAGTTGGATGGTGGAGAGG		
^1^ *ND6*	F: TAACAACAAACCTCACCCAGCC	NC_053523.1	[27]
	R: GTGTGTCTTTTGCTCGGTTGGA		
^2^ *βactin*	F: ATCCGGACCCTCCATTGTC	NM_205518.1	[27]
	R: AGCCATGCCAATCTCGTCTT		

^1^ Genes used to amplify the fragment of mitochondrial DNA. ^2^ Genes used to amplify the fragment of cDNA (reference genes). Abbreviations: ND4, NADH dehydrogenase subunit 4; COX1, mitochondrial cytochrome c oxidase 1; ATP6, ATP synthase F0 subunit 6; ND6, NADH dehydrogenase subunit 6; F, forward; R, reverse.

**Table 3:** Effect of dietary supplementation of ALA on laying performance and eggshell quality in laying hens under chronic heat stress conditions.

	TN (24 °C)			HS (33 °C)			TN	HS	Pooled SEM	*p*-Value		
ALA (ppm)	0	10	100	0	10	100				Temp.	ALA	T × A
Body weight gain (g/d)	1.62 ^a^	0.81 ^a^	0.86 ^a^	−9.42 ^b^	−6.33 ^b^	−6.54 ^b^	1.10	−7.43	0.714	<0.01	0.74	0.27
Feed intake (g/d)	119.1 ^a^	116.3 ^a^	110.0 ^a^	86.3 ^b^	94.9 ^b^	86.7 ^b^	115.2	89.3	2.216	<0.01	0.06	0.16
^1^ Laying rate (%)	99.6 ^a^	99.5 ^a^	96.8 ^a^	85.9 ^b^	86.7 ^b^	91.1 ^ab^	98.7	88.3	1.097	<0.01	0.04	0.03
^1^ Egg weight (g)	60.6	60.2	60.2	59.0	57.9	58.7	60.3	58.5	0.416	<0.05	0.66	0.64
^1^ Daily egg production (g/d)	60.4 ^a^	59.9 ^a^	58.2 ^ab^	50.7 ^b^	50.4 ^b^	53.6 ^ab^	59.5	51.8	0.863	<0.01	0.08	0.06
^2^ Eggshell strength (kg/cm^2^)	4.09 ^a^	3.95 ^a^	3.9 ^a^	3.19 ^b^	3.92 ^a^	3.61 ^ab^	3.97	3.58	0.068	<0.01	0.15	0.14
^2^ Eggshell weight (g)	6.96 ^a^	6.82 ^a^	6.82 ^a^	5.71 ^b^	6.27 ^b^	6.00 ^b^	6.67	5.99	0.084	<0.01	0.38	0.35
^2^ Eggshell thickness (mm)	0.43 ^a^	0.42 ^a^	0.42 ^a^	0.37 ^b^	0.39 ^b^	0.39 ^b^	0.42	0.39	0.004	<0.01	0.56	0.48

^a,b^ Differences between groups were indicated by different lowercase letters (*p* < 0.05). ^1^ Laying performance refers to the average data of each group of hens (n = 9–10 for each week). ^2^ Eggshell quality refers to the average data of each group of eggs (n = 9–10 for each week). Abbreviations: TN, thermoneutral; HS, heat stress; ALA, 5-aminolevulinic acid; Temp, temperature.

**Table 4:** Effect of dietary supplementation of ALA on plasma biochemical parameters in laying hens under chronic heat stress conditions.

	TN (24 °C)			HS (33 °C)			TN	HS	Pooled SEM	*p*-Value		
ALA (ppm)	0	10	100	0	10	100				Temp.	ALA	T × A
Creatinin (mg/dL)	0.04 ^b^	0.04 ^b^	0.03 ^b^	0.06 ^a^	0.05 ^ab^	0.05 ^ab^	0.04	0.05	0.002	<0.01	0.06	0.80
BUN (mg/dL)	0.78	0.83	0.88	0.73	0.76	0.80	0.83	0.77	0.026	0.45	0.63	0.77
Albmin (mg/dL)	2.49 ^a^	2.45 ^a^	2.39 ^a^	2.13 ^b^	2.24 ^ab^	2.06 ^b^	2.44	2.14	0.033	<0.01	0.26	0.81
Calsium (mg/dL)	28.23 ^ab^	29.27 ^a^	30.87 ^a^	22.37 ^c^	25.93 ^bc^	23.9 ^bc^	29.46	24.12	0.611	<0.01	0.12	0.58
Phosphote (mg/dL)	4.05 ^ab^	4.54 ^a^	4.89 ^a^	3.03 ^b^	3.91 ^ab^	3.27 ^b^	4.49	3.41	0.071	0.02	0.09	0.23

^a–c^ Differences between groups were indicated by different lowercase letters (*p* < 0.05). Plasma contents refer to the average of each hen group. (n = 9 or 10). Abbreviations: TN, thermoneutral; HS, heat stress; ALA, 5-aminolevulinic acid; Temp, temperature; BUN, blood urea nitrogen.

## Data Availability

All data generated or analyzed during this study are available from the corresponding authors on reasonable request.

## Abbreviations

The following abbreviations are used in this manuscript:

ATP6	ATP synthase F0 subunit 6
cGAS	cyclic GMP-AMP synthase
COX1	mitochondrial cytochrome c oxidase 1
HO-1	heme oxygenase-1
IL-1β	interleukin-1β
IRF7	type I interferon regulatory factor 7
Keap1	Kelch-like ECH-associated protein 1
MAVS	mitochondrial antiviral signaling
MDA5	melanoma differentiation-associated gene 5
mtDNA	mitochondrial DNA copy number
ND4	NADH dehydrogenase subunit 4
ND6	NADH dehydrogenase subunit 6
NF-ĸB	nuclear factor-κB
NRF2	nuclear factor E2-related factor 2
ROS	reactive oxygen species
STING	stimulator of interferon genes
TBARS	2-thiobarbituric acid reactive substances
TGF-β	transforming growth factor-β
18srRNA	18S ribosomal RNA

## References

[1] Mirzabaev A., Bezner Kerr R., Hasegawa T., Pradhan P., Wreford A., Cristina Tirado von der Pahlen M., Gurney-Smith H. (2023). Severe Climate Change Risks to Food Security and Nutrition. Clim. Risk Manag..

[2] Godde C.M., Mason-D’Croz D., Mayberry D.E., Thornton P.K., Herrero M. (2021). Impacts of Climate Change on the Livestock Food Supply Chain; a Review of the Evidence. Glob. Food Secur..

[3] Allahverdi A., Feizi A., Takhtfooladi H.A., Nikpiran H. (2013). Effects of Heat Stress on Acid-Base Imbalance, Plasma Calcium Concentration, Egg Production and Egg Quality in Commercial Layers. Glob. Vet..

[4] De Baets R., Buyse K., Antonissen G., Delezie E. (2024). Betaine and Feed Restriction as Potential Mitigation Strategies against Heat Stress in Two Strains of Laying Hens. Poult. Sci..

[5] Blaine J., Chonchol M., Levi M. (2015). Renal Control of Calcium, Phosphate, and Magnesium Homeostasis. Clin. J. Am. Soc. Nephrol..

[6] Nanto-Hara F., Yamazaki M., Murakami H., Ohtsu H. (2023). Chronic Heat Stress Induces Renal Fibrosis and Mitochondrial Dysfunction in Laying Hens. J. Anim. Sci. Biotechnol..

[7] Nanto-Hara F., Ohtsu H. (2024). In Laying Hens, Chronic Heat Stress-Induced Renal Fibrosis Is Potentially Promoted by Indoxyl Sulfate. Sci. Rep..

[8] Kang Z., Zhang J., Zhou J., Qi Q., Du G., Chen J. (2012). Recent Advances in Microbial Production of δ-Aminolevulinic Acid and Vitamin B12. Biotechnol. Adv..

[9] Jiang M., Hong K., Mao Y., Ma H., Chen T., Wang Z. (2022). Natural 5-Aminolevulinic Acid: Sources, Biosynthesis, Detection and Applications. Front. Bioeng. Biotechnol..

[10] Luo Z., Pan F., Zhu Y., Du S., Yan Y., Wang R., Li S., Xu H. (2022). Synergistic Improvement of 5-Aminolevulinic Acid Production with Synthetic Scaffolds and System Pathway Engineering. ACS Synth. Biol..

[11] Pu W., Chen J., Zhou Y., Qiu H., Shi T., Zhou W., Guo X., Cai N., Tan Z., Liu J. (2023). Correction: Systems Metabolic Engineering of Escherichia Coli for Hyper-Production of 5-Aminolevulinic Acid. Biotechnol. Biofuels Bioprod..

[12] Sasaki M., Watanabe T., Tanaka T., Tanaka T. (2002). Biosynthesis, Biotechnological Production and Applications of 5-Aminolevulinic Acid. Appl. Microbiol. Biotechnol..

[13] Soto I.C., Fontanesi F., Myers R.S., Hamel P., Barrientos A. (2012). A Heme-Sensing Mechanism in the Translational Regulation of Mitochondrial Cytochrome c Oxidase Biogenesis. Cell Metab..

[14] Ogura S., Maruyama K., Hagiya Y., Sugiyama Y., Tsuchiya K., Takahashi K., Abe F., Tabata K., Okura I., Nakajima M. (2011). The Effect of 5-Aminolevulinic Acid on Cytochrome c Oxidase Activity in Mouse Liver. BMC Res. Notes.

[15] Ota U., Hara T., Nakagawa H., Tsuru E., Tsuda M., Kamiya A., Kuroda Y., Kitajima Y., Koda A., Ishizuka M. (2017). 5-Aminolevulinic Acid Combined with Ferrous Ion Reduces Adiposity and Improves Glucose Tolerance in Diet-Induced Obese Mice via Enhancing Mitochondrial Function. BMC Pharmacol. Toxicol..

[16] Terada Y., Inoue K., Matsumoto T., Ishihara M., Hamada K., Shimamura Y., Ogata K., Inoue K., Taniguchi Y., Horino T. (2013). 5-Aminolevulinic Acid Protects against Cisplatin-Induced Nephrotoxicity without Compromising the Anticancer Efficiency of Cisplatin in Rats In Vitro and In Vivo. PLoS ONE.

[17] Liu C., Zhu P., Fujino M., Isaka Y., Ito H., Takahashi K., Nakajima M., Tanaka T., Zhuang J., Li X.-K. (2019). 5-Aminolaevulinic Acid (ALA), Enhances Heme Oxygenase (HO)-1 Expression and Attenuates Tubulointerstitial Fibrosis and Renal Apoptosis in Chronic Cyclosporine Nephropathy. Biochem. Biophys. Res. Commun..

[18] Takeda T., Sasai M., Adachi Y., Ohnishi K., Fujisawa J., Izawa S., Taketani S. (2017). Potential Role of Heme Metabolism in the Inducible Expression of Heme Oxygenase-1. Biochim. Biophys. Acta BBA—Gen. Subj..

[19] NARO (2012). Japanese Feeding Standard for Poultry, 2011.

[20] Leary S., Pharmaceuticals F., Underwood W., Anthony R., Cartner S., Johnson C.L., Patterson-Kane E. (2020). AVMA Guidelines for the Euthanasia of Animals: 2020 Edition.

[21] Feng Y., Hu Y., Hou Z., Sun Q., Jia Y., Zhao R. (2020). Chronic Corticosterone Exposure Induces Liver Inflammation and Fibrosis in Association with M6A-Linked Post-Transcriptional Suppression of Heat Shock Proteins in Chicken. Cell Stress Chaperones.

[22] Li M., Raheem M.A., Han C., Yu F., Dai Y., Imran M., Hong Q., Zhang J., Tan Y., Zha L. (2021). The Fowl Adenovirus Serotype 4 (FAdV-4) Induce Cellular Pathway in Chickens to Produce Interferon and Antigen-Presented Molecules (MHCI/II). Poult. Sci..

[23] Wang H., Wang Y., Chai Y., Zhang H., Chang Q., Li J., Zhang R., Bao J. (2024). Prolonged Exposure to a Music-Enriched Environment Mitigates Acute Noise-Induced Inflammation and Apoptosis in the Chicken Spleen by Modulating the Keap-1/Nrf2 and NF-ΚB Pathways. Poult. Sci..

[24] Li Y.P., Bang D.D., Handberg K.J., Jorgensen P.H., Man F.Z. (2005). Evaluation of the Suitability of Six Host Genes as Internal Control in Real-Time RT-PCR Assays in Chicken Embryo Cell Cultures Infected with Infectious Bursal Disease Virus. Vet. Microbiol..

[25] Ohkawa H., Ohishi N., Yagi K. (1979). Assay for Lipid Peroxides in Animal Tissues by Thiobarbituric Acid Reaction. Anal. Biochem..

[26] Lu M.Y., Wang W.W., Qi G.H., Xu L., Wang J. (2021). Mitochondrial Transcription Factor A Induces the Declined Mitochondrial Biogenesis Correlative with Depigmentation of Brown Eggshell in Aged Laying Hens. Poult. Sci..

[27] Zhang X., Wang T., Ji J., Wang H., Zhu X., Du P., Zhu Y., Huang Y., Chen W. (2020). The Distinct Spatiotemporal Distribution and Effect of Feed Restriction on MtDNA Copy Number in Broilers. Sci. Rep..

[28] Hendawy A.O., Khattab M.S., Sugimura S., Sato K. (2020). Effects of 5-Aminolevulinic Acid as a Supplement on Animal Performance, Iron Status, and Immune Response in Farm Animals: A Review. Animals.

[29] Wang J.P., Jung J.H., Kim I.H. (2011). Effects of Dietary Supplementation with Delta-Aminolevulinic Acid on Growth Performance, Hematological Status, and Immune Responses of Weanling Pigs. Livest. Sci..

[30] Wang J.P., Lee J.H., Jang H.D., Yan L., Cho J.H., Kim I.H. (2011). Effects of δ-Aminolevulinic Acid and Vitamin C Supplementation on Iron Status, Production Performance, Blood Characteristics and Egg Quality of Laying Hens: δ-Aminolevulinic Acid and Vitamin C in Laying Hens. J. Anim. Physiol. Anim. Nutr..

[31] Sato K., Matsushita K., Takahashi K., Aoki M., Fuziwara J., Miyanari S., Kamada T. (2012). Dietary Supplementation with 5-Aminolevulinic Acid Modulates Growth Performance and Inflammatory Responses in Broiler Chickens. Poult. Sci..

[32] Hendawy A.O., Shirai M., Takeya H., Sugimura S., Miyanari S., Taniguchi S., Sato K. (2019). Effects of 5-Aminolevulinic Acid Supplementation on Milk Production, Iron Status, and Immune Response of Dairy Cows. J. Dairy. Sci..

[33] Wang Z., Ying Z., Bosy-Westphal A., Zhang J., Schautz B., Later W., Heymsfield S.B., Müller M.J. (2010). Specific Metabolic Rates of Major Organs and Tissues across Adulthood: Evaluation by Mechanistic Model of Resting Energy Expenditure. Am. J. Clin. Nutr..

[34] Bhargava P., Schnellmann R.G. (2017). Mitochondrial Energetics in the Kidney. Nat. Rev. Nephrol..

[35] Miao J., Liu J., Niu J., Zhang Y., Shen W., Luo C., Liu Y., Li C., Li H., Yang P. (2019). Wnt/Β-catenin/RAS Signaling Mediates Age-related Renal Fibrosis and Is Associated with Mitochondrial Dysfunction. Aging Cell.

[36] Bhatti J.S., Bhatti G.K., Reddy P.H. (2017). Mitochondrial Dysfunction and Oxidative Stress in Metabolic Disorders—A Step towards Mitochondria Based Therapeutic Strategies. Biochim. Biophys. Acta—Mol. Basis Dis..

[37] Ho H.-J., Shirakawa H. (2022). Oxidative Stress and Mitochondrial Dysfunction in Chronic Kidney Disease. Cells.

[38] Li H., Shen L., Hu P., Huang R., Cao Y., Deng J., Yuan W., Liu D., Yang J., Gu H. (2017). Aging-Associated Mitochondrial DNA Mutations Alter Oxidative Phosphorylation Machinery and Cause Mitochondrial Dysfunctions. Biochim. Biophys. Acta BBA—Mol. Basis Dis..

[39] Shimura M., Nozawa N., Ogawa-Tominaga M., Fushimi T., Tajika M., Ichimoto K., Matsunaga A., Tsuruoka T., Kishita Y., Ishii T. (2019). Effects of 5-Aminolevulinic Acid and Sodium Ferrous Citrate on Fibroblasts from Individuals with Mitochondrial Diseases. Sci. Rep..

[40] Chung K.W., Dhillon P., Huang S., Sheng X., Shrestha R., Qiu C., Kaufman B.A., Park J., Pei L., Baur J. (2019). Mitochondrial Damage and Activation of the STING Pathway Lead to Renal Inflammation and Fibrosis. Cell Metab..

[41] Maekawa H., Inoue T., Ouchi H., Jao T.M., Inoue R., Nishi H., Fujii R., Ishidate F., Tanaka T., Tanaka Y. (2019). Mitochondrial Damage Causes Inflammation via CGAS-STING Signaling in Acute Kidney Injury. Cell Rep..

[42] Islam M.A., Noguchi Y., Taniguchi S., Yonekura S. (2021). Protective Effects of 5-Aminolevulinic Acid on Heat Stress in Bovine Mammary Epithelial Cells. Anim. Biosci..

[43] Uchida A., Kidokoro K., Sogawa Y., Itano S., Nagasu H., Satoh M., Sasaki T., Kashihara N. (2019). 5-Aminolevulinic Acid Exerts Renoprotective Effect via Nrf2 Activation in Murine Rhabdomyolysis-induced Acute Kidney Injury. Nephrology.

[44] Fujino M., Nishio Y., Ito H., Tanaka T., Li X.K. (2016). 5-Aminolevulinic Acid Regulates the Inflammatory Response and Alloimmune Reaction. Int. Immunopharmacol..

[45] Suzuki T., Takahashi J., Yamamoto M. (2023). Molecular Basis of the KEAP1-NRF2 Signaling Pathway. Mol. Cells.

[46] Tu W., Wang H., Li S., Liu Q., Sha H. (2019). The Anti-Inflammatory and Anti-Oxidant Mechanisms of the Keap1/Nrf2/ARE Signaling Pathway in Chronic Diseases. Aging Dis..

[47] Du D., Lv W., Su R., Yu C., Jing X., Bai N., Hasi S. (2021). Hydrolyzed Camel Whey Protein Alleviated Heat Stress-Induced Hepatocyte Damage by Activated Nrf2/HO-1 Signaling Pathway and Inhibited NF-ΚB/NLRP3 Axis. Cell Stress Chaperones.

[48] Hamada S., Mae Y., Takata T., Hanada H., Kubo M., Taniguchi S., Iyama T., Sugihara T., Isomoto H. (2023). Five-Aminolevulinic Acid (5-ALA) Induces Heme Oxygenase-1 and Ameliorates Palmitic Acid-Induced Endoplasmic Reticulum Stress in Renal Tubules. Int. J. Mol. Sci..

[49] Chen J., Wang H., Wu Z., Gu H., Li C., Wang S., Liu G. (2022). Effects of 5-Aminolevulinic Acid on the Inflammatory Responses and Antioxidative Capacity in Broiler Chickens Challenged with Lipopolysaccharide. Animal.

